# Advances in Molecular Imaging in Infective Endocarditis

**DOI:** 10.3390/vaccines11020420

**Published:** 2023-02-12

**Authors:** Katarzyna Holcman, Paweł Rubiś, Andrzej Ząbek, Krzysztof Boczar, Piotr Podolec, Magdalena Kostkiewicz

**Affiliations:** 1 Department of Cardiac and Vascular Diseases, Jagiellonian University Medical College, John Paul II Hospital, 31-202 Krakow, Poland; 2 Department of Nuclear Medicine, John Paul II Hospital, 31-202 Krakow, Poland; 3 Department of Electrocardiology, Jagiellonian University Medical College, John Paul II Hospital, 31-202 Krakow, Poland

**Keywords:** infective endocarditis, ^18^F-FDG PET/CT, SPECT, scintigraphy, ^18^F-FDG PET/CT

## Abstract

Infective endocarditis (IE) is a growing epidemiological challenge. Appropriate diagnosis remains difficult due to heterogenous etiopathogenesis and clinical presentation. The disease may be followed by increased mortality and numerous diverse complications. Developing molecular imaging modalities may provide additional insights into ongoing infection and support an accurate diagnosis. We present the current evidence for the diagnostic performance and indications for utilization in current guidelines of the hybrid modalities: single photon emission tomography with technetium99m-hexamethylpropyleneamine oxime–labeled autologous leukocytes (^99m^Tc-HMPAO-SPECT/CT) along with positron emission tomography with fluorodeoxyglucose (^18^F-FDG PET/CT). The role of molecular imaging in IE diagnostic work-up has been constantly growing due to technical improvements and the increasing evidence supporting its added diagnostic and prognostic value. The various underlying molecular processes of ^99m^Tc-HMPAO-SPECT/CT as well as ^18^F-FDG PET/CT translate to different imaging properties, which should be considered in clinical practice. Both techniques provide additional diagnostic value in the assessment of patients at risk of IE. Nuclear imaging should be considered in the IE diagnostic algorithm, not only for the insights gained into ongoing infection at a molecular level, but also for the determination of the optimal clinical therapeutic strategies.

## 1. Introduction

Infective endocarditis (IE) is considered to be a challenging disease both for initial evaluation and subsequent treatment due to complex etiopathogenesis and the presentation. Rising rates of cardiac implantable electronic devices (CIED) implantation in the elderly with the rising number of co-morbidities has led to an increase in the prevalence of cardiac device-related IE (CDRIE) cases [1,2,3,4,5]. Moreover, despite efforts towards accurate diagnosis and management, profound advances in microbiological testing, and multimodality imaging, the mortality rates stemming from IE have not decreased in more than two decades [6]. The infection extends to the electrode leads, native and prosthetic valves, and endocardium, forming vegetations composed of fibrin, microorganisms, platelets, and inflammatory cells [2]. As a result of the various causative microorganisms, intracardiac lesions, and coexisting comorbidities, IE may have an uncharacteristic clinical presentation, which hinders proper and timely diagnosis [7]. Signs and symptoms are including cardiac lesions, extracardiac embolic foci, immune-mediated reactions, and heart failure. Currently, there is no single reliable examination that can be conducted to establish a diagnosis and the evaluation includes diagnostic criteria depending on the type of the disease [8]. Since in selected populations, especially with CDRIE, Duke criteria have low diagnostic accuracy, due to high rates of negative microbiological testing results and difficulties in interpreting echocardiographic images with artifacts related to the prosthetic valves and electrodes, there have been in recent years efforts to establish novel IE and CDRIE criteria [2]. Indeed, inappropriate diagnosis may cause detrimental outcomes—inappropriate invasive procedures or delays in treatment [9].

In recent years, there has been growing scientific and clinical data supporting the application of hybrid modalities: positron emission tomography/ computed tomography with fluorodeoxyglucose (^18^F-FDG PET/CT) along with single photon emission tomography/ computed tomography with technetium99m-hexamethylpropyleneamine oxime–labeled autologous leukocytes (^99m^Tc-HMPAO-SPECT/CT) [10]. Developing nuclear imaging techniques may provide additional insights into the ongoing IE and further guide tailored treatment. These imaging methods are characterized by dissimilar diagnostic characteristics and limitations, both arising from the phenomena upon which these modalities are developed. The ^99m^Tc-HMPAO-SPECT/CT is based on the intracellular labeling of isolated white blood cells with ^99m^Tc-HMPAO complex and a protocol including multiple image acquisitions in time specific manner [6,11]. The ^18^F-FDG PET/CT depends on the radiolabeled glucose analog (^18^F-FDG) retention in cells with high numbers of metabolically active glucose transporters expressed on their cell surface, such as, macrophages, leukocytes, and lymphocytes [12,13,14].

There has been growing evidence that evolving nuclear medicine modalities might provide additional diagnostic and prognostic information in this complex and challenging group of patients. In recent years ^99m^Tc-HMPAO-SPECT/CT and ^18^F-FDG PET/CT imaging results were incorporated for the first time in IE diagnostic algorithm and criteria in European Society of Cardiology (ESC) Guidelines, in prosthetic valve IE (PVE) and were deemed an additive method in patients with CDRIE, positive microbiology and non-diagnostic echocardiographic results (Table 1) [2]. This recommendation was broadened in the latest European Heart Rhythm Association (EHRA) document [15]. This consensus introduced the Novel 2019 International CIED Infection Criteria, which are based on nuclear imaging results (Table 2). The inclusion of molecular imaging techniques into the IE diagnostic process improves the appropriate classification of patients and helps to avoid unnecessary treatment.

## 2. ^99m^Tc-HMPAO-SPECT/CT

The hybrid technique ^99m^Tc-HMPAO-SPECT/CT relies upon the intracellular labeling of autologous white blood cells [16]. The HMPAO tracer and ^99m^Tc radioisotope form a lipid-soluble complex, which passes through the cell membrane due to passive diffusion. Radiotracer remains in the cell as a result of the conversion of the complex into a hydrophilic one, by reducing agents such as glutathione, and binding to non-diffusible proteins and cell organelles [17]. White blood cell labeling has been made more accessible after introducing disposable sterile closed devices for the procedure. However, the process still remains time-consuming and necessitates blood handling [18]. Once administered intravenously radiolabeled white blood cells migrate to the respiratory system and, if not damaged, afterward to the liver, the spleen, and the reticuloendothelial tissues [16,17]. Following that, the migration is directed by chemotactic attraction to the bone marrow and infected sites [16,17]. Thus, ^99m^Tc-HMPAO-SPECT/CT, is performed according to a 24-h-long protocol, including early (30–60 min), delayed (2–4 h), and late (20–24 h) acquisitions [16]. The accumulation may be influenced by antibiotic therapy, the type of pathogen, and the vascularization of the infected tissue [17]. Nevertheless, this technique provides high specificity, especially in the context of differentiating sterile and infectious morphological intracardiac lesions [19].

Autologous white blood cells can be radiolabeled ex-vivo using ^99m^Tc-HMPAO or ^111^In-oxine. There is stronger evidence and wider application of ^99m^Tc-HMPAO compared to ^111^In-oxine in this particular indication. Firstly, scintigraphy performed with ^111^In has poorer image quality and significantly higher radiation dose due to the long half-life time (6 h vs. 2.8 days) [16]. Moreover, a white blood cell scan performed with ^99m^Tc-HMPAO has higher sensitivity and specificity compared to ^111^In-oxine (60–85% and 78–94%, for ^111^In-oxine; 96 and 92% for ^99m^Tc-HMPAO) which has been noted in European Association of Nuclear Medicine procedural guidelines [16].

The ^99m^Tc-HMPAO-SPECT/CT examination is deemed as positive for IE in the presence of at least one intracardiac and/or in the vicinity of the CIED electrode site of increased radiotracer uptake, and which is characterized by a time-dependent radioactivity pattern. Due to the specific characteristics of leucocyte biodistribution over time, it is possible to differentiate foci of increased tracer uptake which are diagnostic for infection [16,17]. White blood cells are observed in various organs and regions of bone marrow at specific points in time after their intravenous administration; however, the intensity of physiological uptake does not increase over time—in the delayed and late ^99m^Tc-HMPAO-SPECT/CT acquisitions. In contrast, leucocytes display signs of accumulation in the infectious sites over a period of time. Therefore, it is crucial to evaluate lesions within a cardiovascular system suspected of IE in a time-dependent manner, including early, delayed, and late images.

**Figure 1:**
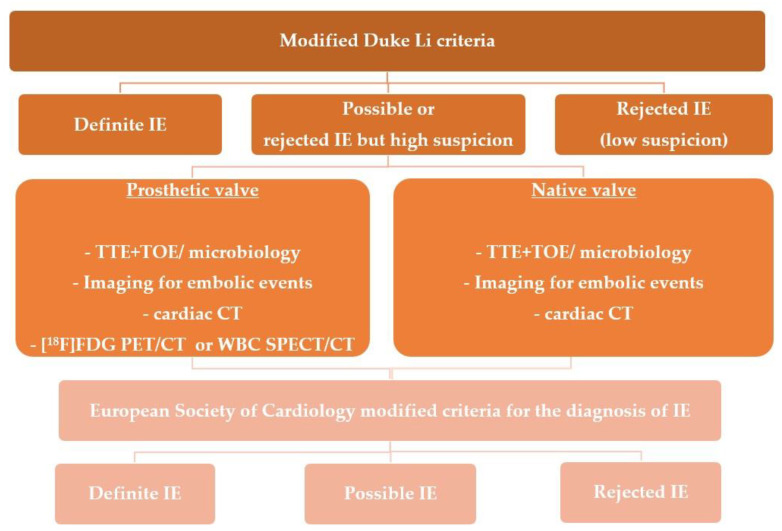
Shows indications for molecular imaging techniques in patients with suspected infective endocarditis. Adapted from Habib, G.; et al. ESC Guidelines for the management of infective endocarditis [2]. CT—computed tomography; Abbreviations are listed in Table 1 and Table 2 legends. TOE–transesophageal echocardiography; TTE—transthoracic echocardiography.

**Figure 2:**
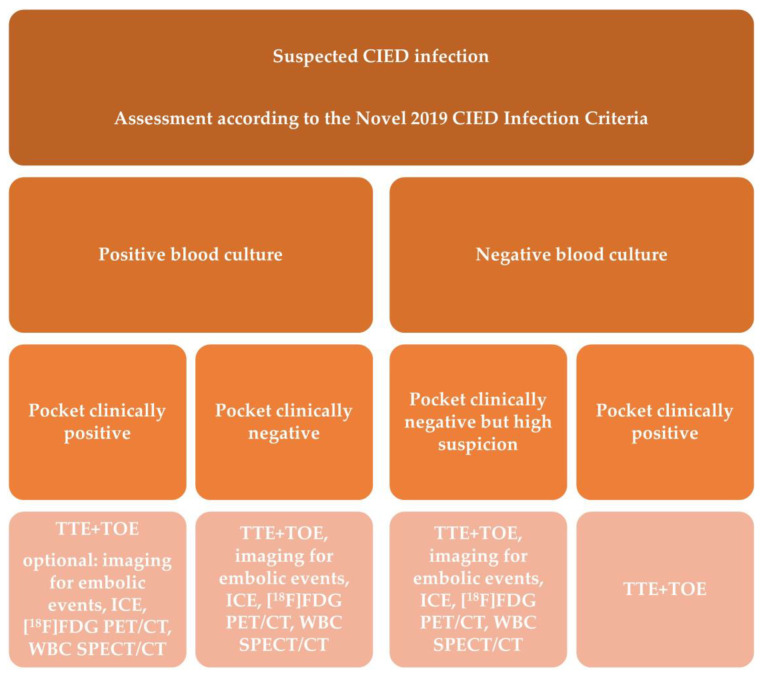
Shows indications for molecular imaging techniques in patients with suspected cardiac device-related infective endocarditis. Adapted from Blomström-Lundqvist, C.; et al. European Heart Rhythm Association (EHRA) international consensus document on how to prevent, diagnose, and treat cardiac implantable electronic device infections-endorsed by the Heart Rhythm Society (HRS), the Asia Pacific Heart Rhythm Society (APHRS), the Latin American Heart Rhythm Society (LAHRS), International Society for Cardiovascular Infectious Diseases (ISCVID) and the European Society of Clinical Microbiology and Infectious Diseases (ESCMID) in collaboration with the European Association for Cardio-Thoracic Surgery (EACTS) [15]. Abbreviations are listed in Table 1 and Table 2 and Figure 1 legends.

Recent years have yielded more data justifying the application of this modality in IE evaluation, it was included for the first time in the recent ESC Guidelines within the diagnostic algorithm for PVE and in EHRA consensus in selected clinical scenarios-suspected CIED infection and coexisting positive blood cultures and negative echocardiography, for assessment of embolic events and in course of persistent sepsis after the procedure of device extraction (Figure 1 and Figure 2) [2,15]. In clinical practice distinction between CDRIE, including cardiac tissues and/or the intravascular portion of the lead and local device infection (LDI), limited solely to the CIED lodge, is crucial in terms of differences in regard to prognosis and treatment. Patients with CDRIE have an increase of 15–20% in mortality after 1 year and require a prolonged course of intravenous antibiotic therapy combined with complete hardware removal [2,4,20,21].

The ^99m^Tc-HMPAO-SPECT/CT examination has high specificity and sensitivity in suspected PVE, especially in case of inconclusive transthoracic echocardiography (TTE) [22,23,24]. The diagnostic accuracy of this technique was validated based on histopathological examination as a reference gold standard [25]. The ^99m^Tc-HMPAO-SPECT/CT supports the more accurate reclassification of patients with suspected PVE [25]. Moreover, it has excellent diagnostic value in the visualization of perivalvular abscesses [25]. As a result, ^99m^Tc-HMPAO-SPECT/CT was included in IE diagnostic criteria and algorithm in ESC Guidelines in patients with suspected PVE [2]. The diagnostic parameters in solely the NVE group have not been validated so far [2].

The diagnostic properties of ^99m^Tc-HMPAO-SPECT/CT in cardiac device-related infections were evaluated in multiple studies [26,27,28,29,30]. It is characterized cumulatively by 60–93.7% sensitivity, 88–100% specificity, 84.6–93.9% negative predictive value (NPV), and 74–100% positive predictive value (PPV) [31]. Besides the extent of device involvement, the detected extracardiac inflammatory lesions were diagnosed as: extracardiac concomitant infection, initial sites of infection leading to CDRIE, or coexisting inflammatory lesions [31]. The ^99m^Tc-HMPAO-SPECT/CT examination is able to reliably exclude CDRIE in patients with fever or sepsis (95% NPV) [27]. The inclusion of the ^99m^Tc-HMPAO-SPECT/CT positive result as a major criterion substantially improves the initial Duke-Li classification [24,26]. Moreover, in CDRIE suspicion, positive ^99m^Tc-HMPAO-SPECT/CT results are associated with increased rates of in-hospital mortality (11.4% vs. 0%, respectively; odds ratio: 19.6; 95% confidence interval [CI]: 1.02 to 374.70), complications (43% vs. 9%, respectively; hazard ratio [HR]: 5.9; 95% CI: 2.27 to 15.20), and procedure of a hardware removal (57% vs. 16%, respectively; HR: 4.3; 95% CI: 2.07 to 19.08) [30].

**Figure 3:**
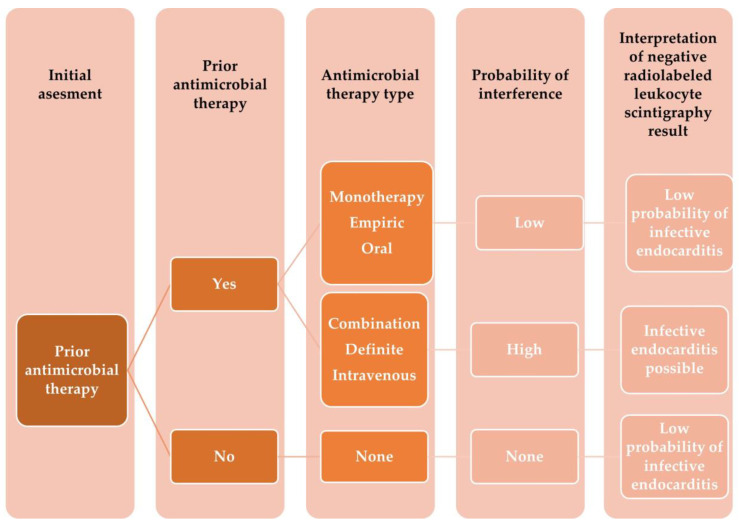
Shows the proposed algorithm on how to interpret a negative ^99m^Tc-HMPAO-SPECT/CT results in patients with suspected infective endocarditis receiving antimicrobial therapy prior to examination. Adapted from Holcman, K.; et al. To what extent does prior antimicrobial therapy affect the diagnostic performance of radiolabeled leukocyte scintigraphy in infective endocarditis [32]?

**Figure 4:**
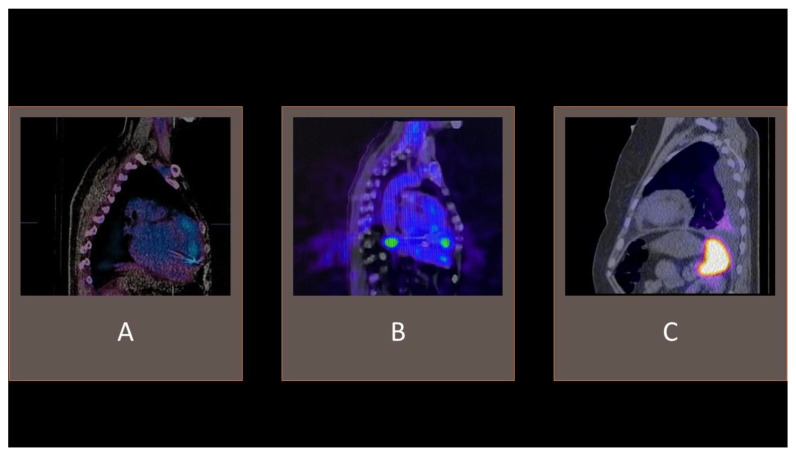
Examples of ^99m^Tc-HMPAO-SPECT/CT. (**A**) tracer uptake consistent with cardiac device-related infective endocarditis with an accumulation of radiolabeled leucocytes in the vicinity of an implanted electrode. (**B**) visible tracer uptake in the vicinity of an implanted left ventricle assist device. (**C**) accumulation of radiolabeled leucocytes in the pleural cavity effusion, without pathological intracardiac uptake.

In a prospective study assessing whether prior antimicrobial treatment impacts the diagnostic profile of this modality in IE diagnosis, this technique displayed 81.95% accuracy, 86.92% specificity, 73.33% sensitivity, 84.96% NPV, and 76.39% PPV [32]. Importantly, antimicrobial therapy was related to higher rates of false-negative ^99m^Tc-HMPAO-SPECT/CT results (OR, 4.63; 95% CI, 1.41 to 15.23, *p* = 0.01). Given that prolonged pharmacotherapy is one of the crucial elements in managing patients with IE, it is vital to understand its impact on the diagnostic tools (Figure 3 and Figure 4).

## 3. ^18^F-FDG PET/CT

The ^18^F-FDG PET/CT, relies on the radiotracer accumulating in cells with high numbers of metabolically active glucose transporters expressed on their cell surface—activated inflammatory cells such as macrophages and lymphocytes. Administered ^18^F-FDG passes to the cell via glucose transporters (GLUTs), subsequently is phosphorylated by hexokinases (HXKs) to FDG-6-phosphate, afterward remaining in the cell [33]. The recommended administered activity is at a level of 2.5–5 MBq/kg, which corresponds with 175 to 350MBq for an adult weighing 70 kg [34]. The acquisition should be performed 60 min after the radiotracer administration [35]. The ^18^F-FDG PET/CT images should be evaluated in 2D planes and in 3D maximum intensity projection cine mode, in terms of intensity and the pattern of the uptake. The heterogeneous uptake can be associated with an infection [36]. Moreover, ^18^F-FDG PET/CT provides quantification opportunities and extracardiac septic foci assessment [37,38].

The diagnostic accuracy of this technique is depending on the proper suppression of the natural radiotracer myocardial uptake by means of a low-carbohydrate and high-fat diet, followed by a period of fast [35]. Although the acquisition itself is short, this technique requires time-consuming patient preparation. The Society of Nuclear Medicine and Molecular Imaging (SNMMI)/American Society of Nuclear Cardiology (ASNC)/Society of Cardiovascular CT (SCCT) guidelines advise a fat-enriched diet without carbohydrates for 12–24 h prior to the examination, a fast of 12–18 h, followed by the administration of intravenous heparin 15 min prior to radiotracer administration [39]. Nonetheless, it should be noted that in many published studies there are applied variable times of dietary restrictions and pharmacological procedures for study preparation [31]. Those various myocardial suppression protocols ought to be acknowledged in the context of discrepancies in ^18^F-FDG PET/CT diagnostic accuracy in IE work-up. Unification of imaging protocols and standardization of procedures is of paramount importance for providing reliable data for continuous evaluation and further development of those techniques. Moreover, numerous lesions may imitate IE-like uptake—primary and metastatic cardiac tumors, vasculitis, inflammation related to surgical procedures, and foreign body reactions—typically in patients with PVE, as a result of the use of a local tissue adhesive [35].

Currently ^18^F-FDG PET/CT results are included in PVE diagnostic criteria and algorithm, as well as in the Novel 2019 International CIED Infection Criteria [2,15]. This modality is recommended in patients with suspected CDRIE, positive blood cultures, and negative echocardiography, as well as for identification of extracardiac foci [15]. Additionally, it should be performed in the case of *S. aureus* bacteremia in patients with CIED, and for identification of the infection portal of entry [15].

Based on meta-analysis results ^18^F-FDG PET/CT has rather limited pooled sensitivity for the NVE diagnosis, while pooled specificity is high—36% and 99%, respectively [40]. The low spatial resolution of PET/CT imaging reaching 5 mm is considered to be an important limitation for the detection of small vegetations with continuous cardiac movements [41]. Nonetheless, the diagnostic performance of this modality in PVE evaluation was investigated in a meta-analysis including 15 studies with 333 cases, confirming high pooled sensitivity and specificity were at levels of 86% and 84%, respectively [42]. The ^18^F-FDG PET/CT supports the more accurate reclassification of patients with suspected PVE [43,44]. Moreover, a simultaneous acquisition with electrocardiogram-gated CT angiography (CTA) should be considered for increasing anatomical resolution [45]. However, a recent study including patients with all types of disease evaluating a 4-point scoring system showed the sensitivity, specificity, PPV, NPV, and accuracy of the qualitative approach were 93%, 81%, 84%, 91%, and 87%, respectively [46].

There are numerous studies evaluating the diagnostic properties of ^18^F-FDG PET/CT in CDRIE diagnosis [47,48,49,50,51,52,53,54,55,56,57,58,59]. This modality is characterized cumulatively by 86.67–93% accuracy, 62.5–100% specificity, 30.8–100% sensitivity, 66–100% PPV, and 75–100% NPV for CDRIE diagnosis [31]. However, ^18^F-FDG PET/CT presents 86.6% accuracy, 72.2–84.2% sensitivity, 95.6–100% specificity, 86.7–94.1% PPV, 88.9–89.6% NPV while detecting LDI [31]. Importantly, false-negative imaging results occurred in patients who had undergone previous antimicrobial therapy [51,54,55]. Assessment of the maximum standardized uptake values (SUVmax) may provide additional insight into ongoing infections [47,49,50,51,52,53,54,55,56,59]. Results support mainly SUVmax assessment for device pocket infection diagnosis. The value of SUVmax of the lodge was significantly higher in the case of CIED-related infection compared to the control group (4.8 ± 2.4 vs. 2.0 ± 0.8, *p* < 0.001) [47]. Moreover, in patients with microbiologically confirmed lodge involvement, there was a substantial increase of mean local SUVmax of delayed acquisition compared to standard one (4.73 ± 1.72 vs. 3.51 ± 1.92, *p* = 0.0002), followed by a mean lead SUVmax significant increase compared to standard acquisition in patients with infected leads (3.25 ± 0.93 vs. 1.11 ± 1.70, *p* = 0.01) [52]. Moreover, the identification of patients with a “Cold Closed Pocket” (i.e., a negative CIED pocket in ^18^F-FDG PET/CT without skin erosion/perforation) may be clinically relevant, since this subset of patients presents worse long-term survival [55].

## 4. Limitations

There are clear limitations with respect to both modalities. The ^99m^Tc-HMPAO-labeled white blood cell scintigraphy requires multiple acquisitions, blood handling and is contraindicated in pregnancy. The quality of the imaging is limited by neutropenia, small lesions, and the spatial resolution of the scanner. On the other hand, ^18^F-FDG PET/CT results may be impacted by factors such as: the non-optimal suppression of myocardium, artifacts resulting from respiratory movements, inflammatory process, the small size of the assessed structures, and high blood sugar level. These dissimilar underlying molecular mechanisms and acquisition protocols give rise to the various characteristics of those modalities (Table 3). Furthermore, discussed studies are limited by the absence of control groups, their retrospective protocol, inconstancy in relation to the gold standard, and acquisition protocol applied. In the future, these data should be investigated in further prospective multi-center studies.

Echocardiography is the technique of choice for the initial assessment, and doubtlessly plays a key role in the imaging-based management and monitoring of these patients [2]. It is recommended in all subtypes of IE as a first-line imaging technique by current guidelines (Figure 1 and Figure 2) [2,15]. Still, a properly performed echocardiogram does not exclude infection, and additional echocardiographic lesions in patients without an active infective process are frequently observed. Therefore, in such a complex group of patients, with numerous comorbidities, there may be factors present hindering echocardiographic interpretation. Nevertheless, there are many aspects to echocardiography that have made this technique a method of choice in IE diagnostics—accessibility, low cost, high safety profile with no radiation, and the capacity for both anatomical and functional cardiac assessment [60]. In direct comparison, ^99m^Tc-HMPAO-SPECT/CT yields a smaller number of false-positive results [19].

## 5. Future Research Directions

The role of molecular imaging in IE diagnostic work-up has been constantly growing due to technical improvements and the increasing evidence supporting its added diagnostic and prognostic value. Artificial intelligence and machine learning are expanded in the field of molecular imaging for data-driven noise reduction strategies, automated lesion delineation, advanced quantification [35]. Although, there have not been yet published data on artificial intelligence and machine learning potential applications in IE imaging, their role in the future may increase.

Antibiotic-derived PET tracers are currently being investigated and developed to overcome limitations and enhance the specificity (Table 4). More infection-specific radiopharmaceuticals targeting direct interactions with microorganisms may translate into the further enhancement of IE diagnosis. The majority of the radiotracers are currently in the preclinical stages of development; they may be divided into two subgroups: structurally modified and unaltered antibiotic radiotracers [61]. Since most antibiotics are small organic compounds, they are of high value for radiochemical utilization. Despite the fact, that there are numerous agents being currently developed, there is limited data on their clinical applications. Clinical studies in human subjects have shown [^11^C]trimethoprim accuracy in diagnosing biopsy-proven methicillin-sensitive *S. aureus* (MSSA) bone infection [61], [^18^F]F-ciprofloxacin ability to mark soft-tissue infections [62], [^18^F]F-fleroxacin capability to image bronchitis [63]. Although, there were no significant differences observed in [^11^C]erythromycin between healthy and pneumonic lung tissue [64]. Imaging with [^11^C]rifampin in patients with tuberculosis has been shown to be able to discriminate accurately affected pulmonary lesions, which has not been confirmed in terms of brain tissue [65,66]. Although there have been no published data yet on antibiotic-derived PET tracers’ potential application in IE work-up, their role in the future may increase and translate into clinical practice. Undoubtedly, there is a need for multicenter and prospective trials to continue to develop molecular imaging in the area of cardiovascular-related infections.

## 6. Conclusions

Accurate IE diagnosis and multimodality assessment play a crucial role in choosing the best therapeutic approach, which may be involving prolonged antibiotic treatment, cardiac surgery, and complete hardware removal. Since IE has numerous severe complications as well as remains associated with high in-hospital and long-term mortality, thus evolving diagnostic modalities is pivotal for improving outcomes. Nuclear medicine imaging techniques provide additional insight into ongoing IE on a molecular level. The ^99m^Tc-HMPAO-SPECT/CT and ^18^F-FDG PET/CT yield further diagnostic and prognostic value in the assessment of patients at risk of IE, thus should be considered in the IE diagnostic process, according to current indications based on international guidelines. The inclusion of ^99m^Tc-HMPAO-SPECT/CT and ^18^F-FDG PET/CT into the IE diagnostic process improves the appropriate classification of patients and helps to avoid unnecessary treatment, as well as provides additional information about extracardiac lesions. Although, there have not been yet published data on antibiotic-derived PET tracers and artificial intelligence potential application in IE diagnostic process, their role in the future may increase and translate into clinical practice.

## Figures and Tables

**Table 1:** European Society of Cardiology 2015 modified criteria for the diagnosis of infective endocarditis.

Major Criteria
Microbiology
Typical microorganisms from two separate blood cultures (*Viridans streptococci*, *Streptococcus gallolyticus* (*Streptococcus bovis*), HACEK group, *Staphylococcus aureus* or community-acquired enterococci, in the absence of a primary focus). Microorganisms consistent with IE from persistently positive blood cultures (≥2 positive blood cultures of blood samples drawn >12 h apart or all of three or a majority of ≥4 separate cultures of blood (with and last samples drawn ≥1 h apart)). Single positive blood culture for *Coxiella burnetii* or phase I IgG antibody >1:800.
Imaging
Echocardiographic lesions positive for IE: vegetation, abscess, pseudoaneurysm, intracardiac fistula, valvular perforation or aneurysm, new dehiscence of prosthetic valve. Abnormal activity around the site of prosthetic valve implantation detected by ^18^F-FDG PET/CT (if the prosthesis was implanted for >3 months) or radiolabeled leukocytes SPECT/CT. Paravalvular lesions in cardiac CT.
**Minor criteria**
Predisposition of heart condition, or injection drug use. Fever. Vascular lesions: arterial emboli, septic pulmonary infarcts, mycotic aneurysm, intracranial hemorrhage, conjunctival hemorrhages, and Janeway’s lesions. Immunological phenomena: glomerulonephritis, Osler’s nodes, Roth’s spots, and rheumatoid factor. Microbiological evidence: positive blood culture but does not meet a major criterion as noted above or serological evidence of active infection with organism consistent with IE.

CT—computed tomography; FDG—fluorodeoxyglucose; HACEK—*Haemophilus parainfluenzae*, *H. aphrophilus*, *H. paraphrophilus*, *H. influenzae*, *Actinobacillus actinomycetemcomitans*, *Cardiobacterium hominis*, *Eikenella corrodens*, *Kingella kingae*, and *K. denitrificans*; IE—infective endocarditis; Ig—immunoglobulin; PET—positron emission tomography; SPECT—single photon emission computerized tomography. Adapted from Habib, G.; et al. ESC Guidelines for the management of infective endocarditis [2,15].

**Table 2:** The Novel 2019 International CIED Infection Criteria.

Major Criteria
Microbiology
Blood cultures positive for typical microorganisms found in CIED infection and/or IE (Coagulase-negative staphylococci, *S. aureus*). Typical microorganisms from two separate blood cultures (*Viridans streptococci*, *Streptococcus gallolyticus* (*Streptococcus bovis*), HACEK group, *Staphylococcus aureus* or community-acquired enterococci, in the absence of a primary focus). Microorganisms consistent with IE from persistently positive blood cultures (≥2 positive blood cultures of blood samples drawn >12 h apart or all of three or a majority of ≥4 separate cultures of blood (with and last samples drawn ≥1 h apart)). Single positive blood culture for *Coxiella burnetii* or phase I IgG antibody >1:800.
Imaging
Echocardiogram (including ICE) positive for CIED infection: clinical pocket/generator infection or lead-vegetation. Echocardiographic lesions positive for IE: vegetation, abscess, pseudoaneurysm, intracardiac fistula, valvular perforation or aneurysm, new dehiscence of prosthetic valve. [^18^F]FDG PET/CT (caution should be taken in case of recent implants) or radiolabeled WBC SPECT/CT detection of abnormal activity at pocket/generator site, along leads, or at valve site. Paravalvular lesions in cardiac CT.
**Minor criteria**
Predisposition heart condition (e.g., new onset tricuspid valve regurgitation), or injection drug use. Fever. Vascular lesions: arterial emboli, septic pulmonary infarcts, mycotic aneurysm, intracranial hemorrhage, conjunctival hemorrhages, and Janeway’s lesions. Immunological phenomena: glomerulonephritis, Osler’s nodes, Roth’s spots, and rheumatoid factor. Microbiological evidence: positive blood culture but does not meet a major criterion as noted above or serological evidence of active infection with organism consistent with IE or pocket culture or leads culture (extracted by non-infected pocket).

Abbreviations are listed in the Table 1 legend. CIED—cardiac implantable electronic device; ICE—intracardiac echocardiography; WBC—white blood cell. Adapted from Blomström-Lundqvist, C.; et al. European Heart Rhythm Association (EHRA) international consensus document on how to prevent, diagnose, and treat cardiac implantable electronic device infections-endorsed by the Heart Rhythm Society (HRS), the Asia Pacific Heart Rhythm Society (APHRS), the Latin American Heart Rhythm Society (LAHRS), International Society for Cardiovascular Infectious Diseases (ISCVID) and the European Society of Clinical Microbiology and Infectious Diseases (ESCMID) in collaboration with the European Association for Cardio-Thoracic Surgery (EACTS) [15].

**Table 3:** Utilities of ^18^F-FDG PET/CT and ^99m^Tc-HMPAO-labeled white blood cell scintigraphy in infective endocarditis imaging.

	^99m^Tc-HMPAO-SPECT/CT	^18^F-FDG PET/CT
**Protocol—time of acquisitions after tracer injection**	early (30–60 min), delayed (2–4 h), and late (20–24 h) acquisitions	single acquisition—60 min
**Preparation to the study**	isolation of autologous leucocytes, incubation with tracer HMPAO and radioisotope, followed by intravenous autologous radiolabeled leucocytes administration	12–18 h prior to the study fasting, 24 h low-carbohydrate and high-fat diet; additionally intravenous heparin or calcium channel blockers are administered prior to the acquisition
**Dose**	370–740 Megabecquerels	175–350 Megabecquerels
**Quantitative assessment**	limited	possible
**Spatial resolution**	limited	high
**Accessibility**	low	low
**Diagnostic profile**	specific	sensitive
**Prognostic assessment**	predictor of increased in-hospital mortality and complications	‘Cold Closed Pocket’ is related to higher mortality
**Interference from antimicrobial therapy**	yes—related to antimicrobial therapy type	possible—no direct evidence
**Contraindications**	neutropenia, pregnancy	pregnancy, hyperglycemia
**Limitations**	duration and multiple acquisitions blood handling non-specific activity in the bowel (result of hepatic tracer excretion) limited detection of smaller vegetations	non-optimal suppression of myocardial uptake respiratory artifacts non-specific inflammatory lesions difficult to differentiate from infection sites limited detection of smaller vegetations

Abbreviations are listed in Table 1 and Table 2 and Figure 1 legends. HMPAO—hexamethylpropyleneamine-oxime.

**Table 4:** Overview of antibiotic-derived PET radiotracers.

Radiotracer	Evidence
[^11^C]trimethoprim	Clinical data
[^18^F]F-propyl-trimethoprim	Clinical data
[^18^F]F-ciprofloxacin	Clinical data
[^68^Ga]Ga-p-SCN-Bz-NOTA-ciprofloxacin	Preclinical
[^68^Ga]Ga-p-SCN-Bz-DOTA-ciprofloxacin	Preclinical
[^68^Ga]Ga-DOTA-ciprofloxacin	Preclinical
N4′-3-[^18^F]F-propyl-ciprofloxacin	Preclinical
[^18^F]F-lomefloxacin	Clinical data
[^18^F]F-trovafloxacin	Clinical data
[^11^C]isoniazid	Preclinical
2-[^18^F]F-isoniazid	Preclinical
[^11^C]PT70	Preclinical
[^11^C]PT119	Preclinical
[^11^C]pyrazinamide	Preclinical
5-[^18^F]F-pyrazinamide	Preclinical
[^18^F]F-linezolid	Preclinical
[^76^Br]Br-bedaquiline	Preclinical
[^11^C]rifampin	Clinical data
[^11^C]erythromycin	Clinical data

Abbreviations are listed in Table 1 and Table 2 and Figure 1 legends.

## Data Availability

Not applicable.

## References

[1] Kirkfeldt R.E., Johansen J.B., Nohr E.A., Jørgensen O.D., Nielsen J.C. (2013). Complications after cardiac implantable electronic device implantations: An analysis of a complete, nationwide cohort in Denmark. Eur. Heart J..

[2] Habib G., Lancellotti P., Antunes M.J., Bongiorni M.G., Casalta J.-P., Del Zotti F., Dulgheru R., El Khoury G., Erba P.A., Iung B. (2015). 2015 ESC Guidelines for the management of infective endocarditis: The Task Force for the Management of Infective Endocarditis of the European Society of Cardiology (ESC). Endorsed by: European Association for Cardio-Thoracic Surgery (EACTS), the European Association of Nuclear Medicine (EANM). Eur. Heart J..

[3] Greenspon A.J., Patel J.D., Lau E., Ochoa J.A., Frisch D.R., Ho R.T., Pavri B.B., Kurtz S.M. (2011). 16-Year Trends in the Infection Burden for Pacemakers and Implantable Cardioverter-Defibrillators in the United States: 1993 to 2008. J. Am. Coll. Cardiol..

[4] Bongiorni M.G., Kennergren C., Butter C., Deharo J.C., Kutarski A., Rinaldi C., Romano S.L., Maggioni A.P., Andarala M., Auricchio A. (2017). The European Lead Extraction ConTRolled (ELECTRa) study: A European Heart Rhythm Association (EHRA) Registry of Transvenous Lead Extraction Outcomes. Eur. Heart J..

[5] Miro J.M., Ambrosioni J. (2019). Infective endocarditis: An ongoing global challenge. Eur. Heart J..

[6] Habib G., Lancellotti P., Erba P.-A., Sadeghpour A., Meshaal M., Sambola A., Furnaz S., Citro R., Ternacle J., Donal E. (2019). The ESC-EORP EURO-ENDO (European Infective Endocarditis) registry. Eur. Heart J. Qual. Care Clin. Outcomes.

[7] Migaj J., Oko-Sarnowska Z., Kałużna-Oleksy M., Katarzyńska-Szymańska A., Lesiak M., Mitkowski P. (2018). Atypical presentation of cardiac device related infectious endocarditis and complicated follow-up. Pol. Arch. Intern. Med..

[8] Kaura A., Dworakowska D., Dworakowski R. (2017). Infective endocarditis—Cinderella in cardiology. Kardiologia Polska.

[9] Viganego F., O’Donoghue S., Eldadah Z., Shah M.H., Rastogi M., Mazel J.A., Platia E.V. (2012). Effect of Early Diagnosis and Treatment with Percutaneous Lead Extraction on Survival in Patients with Cardiac Device Infections. Am. J. Cardiol..

[10] Erba P.A., Lancellotti P., Vilacosta I., Gaemperli O., Rouzet F., Hacker M., Signore A., Slart R.H.J.A., Habib G. (2018). Recommendations on nuclear and multimodality imaging in IE and CIED infections. Eur. J. Nucl. Med..

[11] Juneau D., Golfam M., Hazra S., Erthal F., Zuckier L., Bernick J., Wells G.A., Beanlands R.S., Chow B.J. (2018). Molecular Imaging for the diagnosis of infective endocarditis: A systematic literature review and meta-analysis. Int. J. Cardiol..

[12] Mahmood M., Abu Saleh O. (2020). The Role of 18-F FDG PET/CT in Imaging of Endocarditis and Cardiac Device Infections. Semin. Nucl. Med..

[13] Kubota R., Yamada S., Kubota K., Ishiwata K., Tamahashi N., Ido T. (1992). Intratumoral distribution of fluorine-18-fluorodeoxyglucose in vivo: High accu-mulation in macrophages and granulation tissues studied by microautoradiography. J. Nucl. Med. Off. Publ. Soc. Nucl. Med..

[14] Ishimori T., Saga T., Mamede M., Kobayashi H., Higashi T., Nakamoto Y., Sato N., Konishi J. (2002). Increased (18)F-FDG uptake in a model of inflammation: Concanavalin A-mediated lymphocyte activation. J. Nucl. Med..

[15] Blomström-Lundqvist C., Traykov V., Erba P.A., Burri H., Nielsen J.C., Bongiorni M.G., Poole J., Boriani G., Costa R., Deharo J.-C. (2019). European Heart Rhythm Association (EHRA) international consensus document on how to prevent, diagnose, and treat cardiac implantable electronic device infections—Endorsed by the Heart Rhythm Society (HRS), the Asia Pacific Heart Rhythm Society (APHRS), the Latin American Heart Rhythm Society (LAHRS), International Society for Cardiovascular Infectious Diseases (ISCVID) and the European Society of Clinical Microbiology and Infectious Diseases (ESCMID) in collaboration with the European Association for Cardio-Thoracic Surgery (EACTS). EP Eur..

[16] Signore A., Jamar F., Israel O., Buscombe J., Martin-Comin J., Lazzeri E. (2018). Clinical indications, image acquisition and data interpretation for white blood cells and anti-granulocyte monoclonal antibody scintigraphy: An EANM procedural guideline. Eur. J. Nucl. Med..

[17] De Vries E.F., Roca M., Jamar F., Israel O., Signore A. (2010). Guidelines for the labelling of leucocytes with 99mTc-HMPAO. Inflammation/Infection Taskgroup of the European Association of Nuclear Medicine. Eur. J. Nucl. Med. Mol. Imaging.

[18] Signore A., Glaudemans A., Malviya G., Lazzeri E., Prandini N., Viglietti A.L., De Vries E.F.J., Dierckx R.A.J.O. (2012). Development and testing of a new disposable sterile device for labelling white blood cells. Q. J. Nucl. Med. Mol. Imaging.

[19] Holcman K., Szot W., Rubiś P., Leśniak-Sobelga A., Hlawaty M., Wiśniowska-Śmiałek S., Małecka B., Ząbek A., Boczar K., Stępień A. (2018). 99mTc-HMPAO-labeled leukocyte SPECT/CT and transthoracic echocardiography diagnostic value in infective endocarditis. Int. J. Cardiovasc. Imaging.

[20] Sohail M.R., Henrikson C.A., Braid-Forbes M.J., Forbes K.F., Lerner D.J. (2014). Increased Long-Term Mortality in Patients with Cardiovascular Implantable Electronic Device Infections. Pacing Clin. Electrophysiol..

[21] Durante-Mangoni E., Ursi M.P., Andini R., Mattucci I., Della Ratta E.E., Iossa D., Bertolino L., De Vivo S., Manduca S., Torella M. (2022). Long-Term Outcome of Infective Endocarditis Involving Cardiac Implantable Electronic Devices: Impact of Comorbidities and Lead Extraction. J. Clin. Med..

[22] Erba P.A., Conti U., Lazzeri E., Sollini M., Doria R., De Tommasi S.M., Bandera F., Tascini C., Menichetti F., Dierckx R.A. (2012). Added Value of ^99m^Tc-HMPAO–Labeled Leukocyte SPECT/CT in the Characterization and Management of Patients with Infectious Endocarditis. J. Nucl. Med..

[23] Hyafil F., Rouzet F., Lepage L., Benali K., Raffoul R., Duval X., Hvass U., Iung B., Nataf P., Lebtahi R. (2013). Role of radiolabelled leucocyte scintigraphy in patients with a suspicion of prosthetic valve endocarditis and inconclusive echocardiography. Eur. Heart J. Cardiovasc. Imaging.

[24] Rouzet F., Chequer R., Benali K., Lepage L., Ghodbane W., Duval X., Iung B., Vahanian A., Le Guludec D., Hyafil F. (2014). Respective Performance of ^18^F-FDG PET and Radiolabeled Leukocyte Scintigraphy for the Diagnosis of Prosthetic Valve Endocarditis. J. Nucl. Med..

[25] Kooshki N., Grambow-Velilla J., Mahida B., Benali K., Nguyen C., Cimadevilla C., Braham W., Pisani A., Iung B., Raffoul R. (2021). Diagnostic performance of White Blood Cell SPECT imaging against intra-operative findings in patients with a suspicion of prosthetic valve endocarditis. J. Nucl. Cardiol..

[26] Calais J., Touati A., Grall N., Laouénan C., Benali K., Mahida B., Vigne J., Hyafil F., Iung B., Duval X. (2019). Diagnostic Impact of ^18^ F-Fluorodeoxyglucose Positron Emission Tomography/Computed Tomography and White Blood Cell SPECT/Computed Tomography in Patients with Suspected Cardiac Implantable Electronic Device Chronic Infection. Circ. Cardiovasc. Imaging.

[27] Erba P.A., Sollini M., Conti U., Bandera F., Tascini C., De Tommasi S.M., Zucchelli G., Doria R., Menichetti F., Bongiorni M.G. (2013). Radiolabeled WBC Scintigraphy in the Diagnostic Workup of Patients with Suspected Device-Related Infections. JACC Cardiovasc. Imaging.

[28] Małecka B.A., Ząbek A., Dębski M., Szot W., Holcman K., Boczar K., Ulman M., Lelakowski J., Kostkiewicz M. (2018). The usefulness of SPECT-CT with radioisotope-labeled leukocytes in diagnosing lead-dependent infective endocarditis. Adv. Clin. Exp. Med..

[29] Holcman K., Małecka B., Rubiś P., Ząbek A., Szot W., Boczar K., Leśniak-Sobelga A., Hlawaty M., Wiśniowska-Śmiałek S., Stępień A. (2019). The role of 99mTc-HMPAO-labelled white blood cell scintigraphy in the diagnosis of cardiac device-related infective endocarditis. Eur. Heart J. Cardiovasc. Imaging.

[30] Holcman K., Rubiś P., Ząbek A., Ćmiel B., Szot W., Boczar K., Wiśniowska-Śmiałek S., Stępień A., Małecka B., Podolec P. (2020). The Prognostic Value of 99mTc-HMPAO-Labeled Leucocyte SPECT/CT in Cardiac Device-Related Infective Endocarditis. JACC Cardiovasc. Imaging.

[31] Holcman K., Rubiś P., Stępień A., Graczyk K., Podolec P., Kostkiewicz M. (2021). The Diagnostic Value of 99mTc-HMPAO-Labelled White Blood Cell Scintigraphy and 18F-FDG PET/CT in Cardiac Device-Related Infective Endocarditis—A Systematic Review. J. Pers. Med..

[32] Holcman K., Rubiś P., Ćmiel B., Ząbek A., Boczar K., Szot W., Kalarus Z., Graczyk K., Hanarz M., Małecka B. (2022). To what extent does prior antimicrobial therapy affect the diagnostic performance of radiolabeled leukocyte scintigraphy in infective endocarditis?. J. Nucl. Cardiol..

[33] Kawada K., Iwamoto M., Sakai Y. (2016). Mechanisms underlying^18^F-fluorodeoxyglucose accumulation in colorectal cancer. World J. Radiol..

[34] Boellaard R., Delgado-Bolton R., Oyen W.J.G., Giammarile F., Tatsch K., Eschner W., Verzijlbergen F.J., Barrington S.F., Pike L.C., Weber W.A. (2015). FDG PET/CT: EANM procedure guidelines for tumour imaging: Version 2.0. Eur. J. Nucl. Med. Mol. Imaging.

[35] Hove D.T., Slart R., Sinha B., Glaudemans A., Budde R. (2021). 18F-FDG PET/CT in Infective Endocarditis: Indications and Approaches for Standardization. Curr. Cardiol. Rep..

[36] Slart R.H.J.A., Glaudemans A.W.J.M., Gheysens O., Lubberink M., Kero T., Dweck M.R., Habib G., Gaemperli O., Saraste A., Gimelli A. (2020). Procedural recommendations of cardiac PET/CT imaging: Standardization in inflammatory-, infective-, infiltrative-, and innervation (4Is)-related cardiovascular diseases: A joint collaboration of the EACVI and the EANM. Eur. J. Nucl. Med..

[37] Memmott M.J., James J., Armstrong I.S., Tout D., Ahmed F. (2015). The performance of quantitation methods in the evaluation of cardiac implantable electronic device (CIED) infection: A technical review. J. Nucl. Cardiol..

[38] Pijl J., Kwee T., Slart R., Glaudemans A. (2021). PET/CT Imaging for Personalized Management of Infectious Diseases. J. Pers. Med..

[39] Dorbala S., Di Carli M.F., Delbeke D., Abbara S., DePuey E.G., Dilsizian V., Forrester J., Janowitz W., Kaufmann P.A., Mahmarian J. (2013). SNMMI/ASNC/SCCT Guideline for Cardiac SPECT/CT and PET/CT 1.0. J. Nucl. Med..

[40] Gomes A., Glaudemans A.W.J.M., Touw D.J., van Melle J.P., Willems T.P., Maass A.H., Natour E., Prakken N., Borra R., van Geel P.P. (2016). Diagnostic value of imaging in infective endocarditis: A systematic review. Lancet Infect. Dis..

[41] Mikail N., Hyafil F. (2021). Nuclear Imaging in Infective Endocarditis. Pharmaceuticals.

[42] Wang T.K.M., Sánchez-Nadales A., Igbinomwanhia E., Cremer P., Griffin B., Xu B. (2020). Diagnosis of Infective Endocarditis by Subtype Using ^18^F-Fluorodeoxyglucose Positron Emission Tomography/Computed Tomography. Circ. Cardiovasc. Imaging.

[43] Saby L., Laas O., Habib G., Cammilleri S., Mancini J., Tessonnier L., Casalta J.-P., Gouriet F., Riberi A., Avierinos J.-F. (2013). Positron Emission Tomography/Computed Tomography for Diagnosis of Prosthetic Valve Endocarditis. J. Am. Coll. Cardiol..

[44] Philip M., Tessonier L., Mancini J., Mainardi J.-L., Fernandez-Gerlinger M.-P., Lussato D., Attias D., Cammilleri S., Weinmann P., Hagege A. (2020). Comparison Between ESC and Duke Criteria for the Diagnosis of Prosthetic Valve Infective Endocarditis. JACC Cardiovasc. Imaging.

[45] Roque A., Pizzi M.N., Fernández-Hidalgo N., Romero-Farina G., Burcet G., Reyes-Juarez J.L., Espinet C., Castell-Conesa J., Escobar M., Ferreira-González I. (2022). The valve uptake index: Improving assessment of prosthetic valve endocarditis and updating [18F]FDG PET/CT(A) imaging criteria. Eur. Heart J. Cardiovasc. Imaging.

[46] Gazzilli M., Albano D., Lucchini S., Peli A., Cerudelli E., Bertagna F., Giubbini R. (2021). New criteria for the diagnosis of infective endocarditis using 18F-FDG PET/CT imaging. J. Nucl. Cardiol..

[47] Salomäki S.P., Saraste A., Kemppainen J., Hurme S., Knuuti J., Nuutila P., Seppänen M., Roivainen A., Airaksinen J., Salo T. (2020). 18F-FDG positron emission tomography/computed tomography of cardiac implantable electronic device infections. J. Nucl. Cardiol..

[48] Rodríguez-Alfonso B., Casanovas M.M., Urda V.C., Marcos M.C., Romero I.S., Ramos-Martínez A. (2020). PET/CT with 18F-FDG in suspected intracardiac device-related infections: Analysis of performance and diagnostic usefulness. Rev. Española Cardiol..

[49] Cautela J., Alessandrini S., Cammilleri S., Giorgi R., Richet H., Casalta J.-P., Habib G., Raoult D., Mundler O., Deharo J.-C. (2012). Diagnostic yield of FDG positron-emission tomography/computed tomography in patients with CEID infection: A pilot study. EP Eur..

[50] Rubini G., Ferrari C., Carretta D., Santacroce L., Ruta R., Iuele F., Lavelli V., Merenda N., D’Agostino C., Sardaro A. (2020). Usefulness of ^18^F-FDG PET/CT in Patients with Cardiac Implantable Electronic Device Suspected of Late Infection. J. Clin. Med..

[51] Jerónimo A., Olmos C., Vilacosta I., Ortega-Candil A., Rodríguez-Rey C., Pérez-Castejón M.J., Fernández-Pérez C., Pérez-García C.N., García-Arribas D., Ferrera C. (2020). Accuracy of 18F-FDG PET/CT in patients with the suspicion of cardiac implantable electronic device infections. J. Nucl. Cardiol..

[52] Leccisotti L., Perna F., Lago M., Leo M., Stefanelli A., Calcagni M.L., Pelargonio G., Narducci M.L., Bencardino G., Bellocci F. (2014). Cardiovascular implantable electronic device infection: Delayed vs standard FDG PET-CT imaging. J. Nucl. Cardiol..

[53] Sarrazin J.-F., Philippon F., Tessier M., Guimond J., Molin F., Champagne J., Nault I., Blier L., Nadeau M., Charbonneau L. (2012). Usefulness of Fluorine-18 Positron Emission Tomography/Computed Tomography for Identification of Cardiovascular Implantable Electronic Device Infections. J. Am. Coll. Cardiol..

[54] Bensimhon L., Lavergne T., Hugonnet F., Mainardi J.-L., Latremouille C., Maunoury C., Lepillier A., Le Heuzey J.-Y., Faraggi M. (2011). Whole body [18F]fluorodeoxyglucose positron emission tomography imaging for the diagnosis of pacemaker or implantable cardioverter defibrillator infection: A preliminary prospective study. Clin. Microbiol. Infect..

[55] Diemberger I., Bonfiglioli R., Martignani C., Graziosi M., Biffi M., Lorenzetti S., Ziacchi M., Nanni C., Fanti S., Boriani G. (2018). Contribution of PET imaging to mortality risk stratification in candidates to lead extraction for pacemaker or defibrillator infection: A prospective single center study. Eur. J. Nucl. Med..

[56] Tlili G., Amroui S., Mesguich C., Riviere A., Bordachar P., Hindié E., Bordenave L. (2015). High performances of 18F-fluorodeoxyglucose PET-CT in cardiac implantable device infections: A study of 40 patients. J. Nucl. Cardiol..

[57] Graziosi M., Nanni C., Lorenzini M., Diemberger I., Bonfiglioli R., Pasquale F., Ziacchi M., Biffi M., Martignani C., Bartoletti M. (2014). Role of 18F-FDG PET/CT in the diagnosis of infective endocarditis in patients with an implanted cardiac device: A prospective study. Eur. J. Nucl. Med..

[58] Ploux S., Riviere A., Amraoui S., Whinnett Z., Barandon L., Lafitte S., Ritter P., Papaioannou G., Clementy J., Jais P. (2011). Positron emission tomography in patients with suspected pacing system infections may play a critical role in difficult cases. Heart Rhythm..

[59] Ahmed F.Z., James J., Cunnington C., Motwani M., Fullwood C., Hooper J., Burns P., Qamruddin A., Al-Bahrani G., Armstrong I. (2015). Early diagnosis of cardiac implantable electronic device generator pocket infection using ¹⁸F-FDG-PET/CT. Eur. Heart J. Cardiovasc. Imaging.

[60] Habib G., Badano L., Tribouilloy C., Vilacosta I., Zamorano J.L., Galderisi M., Voigt J.-U., Sicari R., Cosyns B., Fox K. (2010). Recommendations for the practice of echocardiography in infective endocarditis. Eur. J. Echocardiogr..

[61] Gouws A.C., Kruger H.G., Gheysens O., Zeevaart J.R., Govender T., Naicker T., Ebenhan T. (2022). Antibiotic-Derived Radiotracers for Positron Emission Tomography: Nuclear or “Unclear” Infection Imaging?. Angew. Chem. Int. Ed..

[62] Langer O., Brunner M., Zeitlinger M., Ziegler S., Dobrozemsky G., Lackner E., Joukhadar C., Mitterhauser M., Wadsak W., Minar E. (2004). In vitro and in vivo evaluation of [18F]ciprofloxacin for the imaging of bacterial infections with PET. Eur. J. Nucl. Med..

[63] Babich J.W., Rubin R.H., Graham W.A., Wilkinson R.A., Vincent J., Fischman A.J. (1996). 18F-labeling and biodistribution of the novel fluoro-quinolone antimicrobial agent, trovafloxacin (CP 99,219). Nucl. Med. Biol..

[64] Wollmer P., Pride N.B., Rhodes C.G., Sanders A., Pike V.W., Palmer A.J., Silvester D.J., Liss R.H. (1982). Measurement of Pulmonary Erythromycin Concentration in Patients with Lobar Pneumonia by Means of Positron Tomography. Lancet.

[65] Ordonez A.A., Wang H., Magombedze G., Ruiz-Bedoya C.A., Srivastava S., Chen A., Tucker E.W., Urbanowski M.E., Pieterse L., Cardozo E.F. (2020). Dynamic imaging in patients with tuberculosis reveals heterogeneous drug exposures in pulmonary lesions. Nat. Med..

[66] Tucker E.W., Guglieri-Lopez B., Ordonez A.A., Ritchie B., Klunk M.H., Sharma R., Chang Y.S., Sanchez-Bautista J., Frey S., Lodge M.A. (2018). Noninvasive^11^C-rifampin positron emission tomography reveals drug biodistribution in tuberculous meningitis. Sci. Transl. Med..

